# Histochemical assays of secretory trichomes and the structure and content of mineral nutrients in *Rubus idaeus* L. leaves

**DOI:** 10.1007/s00709-019-01426-7

**Published:** 2019-08-09

**Authors:** Mirosława Chwil, Mikołaj Kostryco

**Affiliations:** Department of Botany and Plant Physiology, University of Life Sciences in Lublin, Akademicka 15, 20-950 Lublin, Poland

**Keywords:** Red raspberry, Glandular trichomes, Cuticle, Stomata, Micromorphology, Anatomy, Ultrastructure, Elements

## Abstract

Leaves of *Rubus idaeus* are a raw material, ingredients of herbal blend, and a source of antioxidants. There are no data concerning histochemistry of trichomes, and little is known about the leaf structure of this species. The aim of this study was to determine the histochemistry of active compounds and the structure of glandular trichomes, micromorphology, anatomy, and ultrastructure of leaves as well as content of elements. To determine the histochemistry of glandular trichomes, different chemical compounds were used. The leaf structure was analysed using light, scanning, and transmission electron microscopes. The content of elements was determined with atomic absorption spectrometry, and the microanalysis of the epidermis ultrastructure was carried out with a transmission electron microscope equipped with a digital X-ray analyser. In glandular trichomes, polyphenols, terpenes, lipids, proteins, and carbohydrates were identified. The main elements in the ultrastructure of the epidermis were Na, Mo, Se, Ca, and Mg. In dry matter of leaves, K, Mg, Ca, P, and Fe were dominant. Infusions from leaves are safe for health in terms of the Cd and Pb concentrations. Leaves can be a valuable raw material. Non-glandular trichomes prevent clumping of mixed raw materials in herbal mixtures.

## Introduction

The genus *Rubus* characterised by extensive morphological diversity comprises many species divided into 12 subgenera (Naruhashi et al. 2002). The complex taxonomy of this genus is based on various methods, e.g. assessment of genetic relationships between taxa with AFLP markers (Miyashita et al. 2015). Currently, the cultivation of *Rubus idaeus* is increasing substantially due to the nutritional value of the fruit, consumer demand, use of the raw material in various branches of industry, and production of new economically profitable varieties recommended in commodity production (Finn et al. 2005; Ali et al. 2011; Konopiński and Żuber 2013).

Plants from the genus *Rubus*, e.g. *R. idaeus*, *R. plicatus*, *R. saxatilis*, and *R. rosifolius*, and their hybrids provide various herbal raw materials (Ribeiro et al. 1986; Tomczyk and Gudej 2005). *Rubi folium* is an important source of a variety of health-enhancing phytocompounds (Gallaher et al. 2006; Grabek-Lejko and Wojtowicz 2014; Costea et al. 2016; Chwil and Kostryco 2018). Leaves of *R. coreanus* contain tannins (ellagic acid, sanguine H-5) and flavonoids (kaempferol, quercetin) (Om et al. 2016). The beneficial effect of *R. idaeus* and *Rubus plicatus* leaf extracts on the human organism is mainly exploited in medicine and food industry. *R. idaeus* leaves exhibit high antioxidant activity (Buřičová et al. 2011; Aybastıer et al. 2013; Chwil and Kostryco 2018). One of the most important antioxidant enzymes contained in this raw material is catalase, which decomposes hydrogen peroxide particles, thus providing protection against negative effects of oxidative stress (Ścibior and Czeczot 2006; Romanowicz and Krzepiłko 2013).

The high efficiency of trace elements such as Cu, Mo, Se, and Zn as cofactors of antioxidant enzymes, e.g. superoxide dismutase, glutathione peroxidase, xanthine oxidase, and sulphite oxidase, involved in scavenging of free radicals and their effects has been confirmed (Pilon et al. 2006; Mason 2007; Ahmad et al. 2015; Wołonciej et al. 2016). In our previous studies, the highest total antioxidant activity in *R. idaeus* leaves determined with the ferric reducing antioxidant power (FRAP) and Folin-Ciocalteu methods was found for the ‘Radziejowa’ cultivar, while the highest radical DPPH pigmentation capacity was detected in the ʽLaszkaʼ cultivar (Chwil and Kostryco 2018).

This indicates that *R. idaeus* leaves are a good source of exogenous antioxidants in the human diet and can be used as a main herbal raw material and a component of various herbal blends (Grabek-Lejko and Wojtowicz 2014).

As reported by Tomaszewski et al. (2014), trichomes in the leaf epidermis in plants from the genus *Rubus* are regarded as one of the most important traits for classification of the representatives of this genus and for determination of taxa at the level of series, sections, and even subgenera. The distribution, size, and shape of trichomes were found to vary between taxa (Upadhyaya and Furness 1998; Tomaszewski et al. 2014; Karley et al. 2015). Tomaszewski et al. (2014) distinguished three types of trichomes in *Rubus* plants: simple eglandular (unbranched) trichomes, eglandular branched trichomes, and very short secretory uniseriate trichomes. Costea et al. (2016) reported the presence of glandular trichomes on both surfaces of the *R. idaeus* epidermis. Despite the large variety of red raspberry cultivars and hybrids, there are insufficient literature data on the structure of the epidermis and leaf anatomy in this species. Therefore, an attempt to complete this knowledge has been undertaken. The present study is a continuation of previous investigations, in which bioactive compounds present in *Rubi folium* from several red raspberry cultivars were described (Chwil and Kostryco 2018).

The structure of the secretory tissue in *R. idaeus* has rarely been studied. Given the wide application of biologically active substances from *Rubus* plants, it is advisable to supplement the knowledge of the location and chemical composition of glandular trichomes. Therefore, an attempt to complete this knowledge has been undertaken. The aim of this study was to determine and compare the histochemistry of active compounds and the structure of glandular trichomes, micromorphology, anatomy, and ultrastructure of leaves as well as content of selected elements in the three *R. idaeus* varieties. The main research hypotheses included (1) histochemistry and (2) micromorphology of glandular trichomes as well as (3) microanalysis of elements at the level of cell ultrastructure. To our knowledge, this is the first study to provide information in the field of histochemical analyses of trichomes and microanalysis of elements at the level of cell ultrastructure in *Rubus*. We hope that the research results will complement knowledge in this area. The structural traits of leaves may serve as auxiliary taxonomic indications, while the biologically active metabolites may be valuable components of functional foods and may be used in phytotherapy.

## Material and methods

### Research material

Leaves of three *Rubus idaeus* L. cultivars, ‘Glen Ample’, ‘Laszka’, and ‘Radziejowa’, were collected from shrubs growing in a plantation located in Blinów II, south-eastern Poland (50° 52′ 57.03″ N; 22° 23′2 .663″ E). Six samples of young, healthy, and well-developed leaves of each cultivar were taken from the fifth node at the onset of plant flowering. The content of micro- and macroelements as well as trace elements was determined in dry plant material. The collected leaves were dried in a natural air-drying room protected from solar radiation. Hand-made cross sections of fresh leaves were prepared for histological assays, and fragments of laminas were sampled and fixed for microscopic observations aimed at comparison of the structure of trichomes and leaf tissues.

### Fixation of samples and preparation of slides

Fragments were collected from the central and apical part of fresh leaves and fixed in 4% glutaraldehyde for 6 h at room temperature and in 0.1 M phosphate buffer (pH 7.0) at 4 °C for 48 h. To prepare semi-thin sections, fixed samples were rinsed in phosphate buffer and dehydrated for 15 min in a series of ethyl alcohol at concentrations of 15, 30, 50, 70, 90, and 96 and twice in absolute ethanol. Dehydrated plant fragments were embedded in Spurr Low Viscosity resin and polymerised at a temperature of 60 °C for 48 h. Semi-thin cross sections were made from the resin-embedded material. 1-μm-thick sections were cut with a glass knife using a Reichert Ultracut S microtome and stained with 1% toluidine blue and 1% azure II (1:1) at 60 °C for 5 min using modified preparatory procedures (Stadtländer 2007). Stained slides were dried after rinsing with distilled water and 5% ethyl alcohol. Periodic acid-Schiff (PAS) reactions were performed to determine the presence of polysaccharides in the cell walls (Mowry 1963).

Comparative analyses of the epidermis micromorphology and the structure of trichomes and leaf tissues of the studied cultivars were carried out using bright-field light microscopy (LM), fluorescence microscopy (FM), and electron scanning (SEM) microscopy.

### Microscopy

#### Fluorescence microscope

Hand-made cross sections were prepared from fresh leaf material in order to determine groups of compounds contained in the glandular trichomes and analyse the cuticle layer on the surface of the glandular trichomes and epidermis cells. The sections were placed in a drop of a fluorochrome (0.01% auramine O) and embedded in a 50% glycerol solution (Heslop-Harrison and Shivanna 1977). Observations were carried out in a Nikon Eclipse 90i fluorescence microscope equipped with an FITC filter (excitation light 465–495 nm) and a barrier filter (wavelength 515–555 nm).

#### Bright-field light microscope

Comparative analyses of epidermis cells and assimilation mesophyll in the leaves of the three *Rubus idaeus* cultivars were carried out on hand-cut and semi-thin preparations. Microscopic observations and photographic documentation were made under a Nikon Eclipse 400 bright-field light microscope.

#### Scanning electron microscope

After dehydration in an acetone series at concentrations of 15, 30, 50, 70, 90, and 99.5% (anhydrous acetone was used twice), fixed plant samples were critical-point-dried in liquid CO_2_ in an EmiTech K850 dryer. Next, the samples were coated with gold using an EmiTech K550X sputter coater. Observations of the leaf epidermis surface and photographic documentation were made using a Tescan VEGA II LMU scanning electron microscope.

#### Transmission electron microscopy

Fixed leaf fragments were contrasted in a 1.5% osmium tetraoxide solution in order to prepare semi-thin and ultrathin sections. After rinsing with distilled water, 0.5% aqueous uranyl acetate was applied for 2 h at room temperature. When double rinsed with distilled water, fragments of nectaries were dehydrated for 15 min in a series of the following concentrations of ethyl alcohol: 15, 30, 50, 70, 90, 96, and 99.8%, and twice in absolute ethanol. The dehydrated samples were embedded in Spurr Low Viscosity resin and polymerised at a temperature of 60 °C for 48 h. Ultrathin 70-nm-thick sections were cut with a glass knife using a Reichert Ultracut S microtome. Next, they were stained for 40 min with an 8% solution of uranyl acetate in 0.5% acetic acid using modified preparatory procedures (Stadtländer 2007). When double rinsed with distilled water (10 min), the Reynolds reagent was applied for 15 min (Reynolds 1963). After rinsing with water, the sections were dried. The ultrastructure of the nectary cells was viewed under a high-resolution transmission electron microscope (TEM, FEI, USA; Tecnai Spirit G^2^).

### Histochemistry

The main groups of bioactive compounds present in the glandular trichomes in the epidermis of fresh *Rubus idaeus* ‘Laszka’ and ‘Radziejowa’ leaves were stained with relevant histochemical assays using the following chemical compounds: Nadi reagent (naphthol and dimethyl-paraphenylene diamine) for terpenoids and essential oils (David and Carde 1964), Sudan Red 7B (Brundrett et al. 1991) and Sudan Black B (Lison 1960) for total lipids, Nile blue A (Jensen 1962) for neutral and acidic lipids, Lugol’s solution for proteins (Jensen 1962), Fehling’s reagent (Ayoola et al. 2008) for total sugars, PAS reagent (Feder and O'brien 1968) for polysaccharides, and iron chloride (Johansen 1940) and potassium dichromate (Gabe 1968) for phenolic compounds.

### Microanalysis of elements in selected parts of epidermis cells

The qualitative and quantitative microanalysis of the content of selected elements N, P, K, Na, Ca, Mg, S, B, Fe, Zn, Se, Mo, Cd, and Pb in the cuticle and other parts of the cell wall as well as the cytoplasm and vacuoles of the leaf epidermis in *Rubus idaeus* ‘Glen Ample’, ‘Laszka’, and ‘Radziejowa’ was performed with the use of ultrathin non-contrasted sections (thickness 100 nm). The microanalysis was carried out with a high-resolution transmission electron microscope JEM 1400 (JEOL Co., Japan 2008) and a digital microscope controlled from the Windows XP platform and equipped with an X-ray microanalyser (EDS INCA Energy TEM, Oxford Instruments, UK) and an 11-megapixel TEM Morada G2 camera (EMSIS GmbH, Germany).

### Determination of elements

Six samples of young, healthy, and well-developed leaves of each cultivar were taken from the fifth node at the onset of plant flowering. Immediately after sampling, the leaves were dried in a natural air-drying room protected from solar radiation. The dried samples were ground in an analytical mill. 0.5-g aliquots of milled plant samples were transferred to Teflon tubes, and 10 cm^3^ of HNO_3_ was added. Next, the plant material was mineralised in a CEM Mars Xpress microwave oven at a temperature of 210 °C and under pressure of 7 atm. The mineralizates were quantitatively transferred into 50-cm^3^ volumetric flasks and diluted with demineralised water (conductivity of 0.055 μS/cm) to the indicated volume.

The solutions were analysed using a Varian SpectrAA 20FS flame atomic absorption spectrophotometer with the following settings (absorption, slit width, lamp current) for the selected elements, respectively: Ca (422.7 nm, 0.5 nm, 10 mA), Mg (202.6 nm, 1 nm, 4 mA), Fe (248.3 nm, 0.2 nm, 5 mA), Cu (324.8 nm, 0.5 nm, 4 mA), and Zn (213.9 nm, 1 nm, 5 mA). In turn, the emission and slit width was 589 nm and 0.2 nm for Na and 589 nm and 0.2 nm for K, respectively; the HCL lamp was not used for both these elements. A 100-mm slit burner based on a stoichiometric acetylene/air mixture was used as an atomiser. To avoid ionization of Ca-, Mg-, Na-, and K-containing samples and to ensure appropriate atomisation conditions, Schinkel spectral buffer (10 g/dm^3^ CsAl + 100 g/dm^3^ La) was used at a level of 10% of the sample dose.

To determine Pb and Cd, the solutions were analysed using an inductively coupled plasma mass spectrophotometer (ICP Mass Spectrometer Varian MS-820). Argon with a purity of 99.999% was the plasma-forming gas. No reaction chamber (CRI) was used in the analysis. The following parameters were adopted: plasma flow—16 dm^3^/min, nebuliser flow—0.98 dm^3^/min, RF power—1.38 kW, and sampling depth—6.5 mm, and ^114^Cd, ^206^ Pb, ^207^Pb, and ^208^Pb isotopes were used.

The determination was carried out with the standard curve method with deuterium lamp background correction. Ultra Scientific standards with a purity of 99.999% were used for the analysis.

The content of total phosphorus in air-dried leaves of the three *R. idaeus* cultivars was determined with the spectrophotometric method in accordance with the PN-ISO 6491:2000 standard. Samples of ground plant material (2.5 g) were mixed with 1 g of calcium carbonate and incinerated in an electric muffle furnace at 550 °C. The ash was transferred to a 250-ml beaker with 20–50 ml of water. Hydrochloric acid was added until effervescence ceased and additional 10 ml of hydrochloric acid was added. The beaker was placed in a sand bath and evaporated to dryness to obtain an insoluble silica form. After cooling and addition of 10 ml of nitric acid (V), the residue was boiled for 5 min in the sand bath. The liquid was transferred to a 500-ml volumetric flask and filtered. A filtrate aliquot was diluted with water to obtain a solution with a concentration not exceeding 40 μg/ml. Ten millilitres of the solution was transferred to a test tube with the addition of 10 ml of vanadate-molybdate reagent. A portion of the solution was transferred to a measuring cuvette, and absorbance was measured spectrophotometrically at a wavelength of 430 nm against a reference solution. A calibration curve was made by plotting absorbance against the corresponding concentrations of standard phosphorus solutions.

### Morphometric analyses

The length and width as well as the surface area of the secretory head and stalk of glandular trichomes were measured in the adaxial epidermis of the leaves from the three cultivars. In the abaxial epidermis, the size of stomata, i.e. their length and width, as well as the thickness of the cuticular ledge and the length of the groove between the cuticular ledges was compared. The height and width of epidermal cells as well as the size of palisade parenchyma cells in the subcuticular layer were measured on both surfaces of the leaf epidermis in the examined cultivars. The thickness of the lamina and the palisade and spongy parenchyma was compared. The morphometric measurements of the epidermis and tissue structures were performed using LM (Nikon Eclipse 90i) equipped with a computer program for microscopic image analysis (Nikon NIS-Elements version 3.0, Advance Research).

### Statistical analysis of the research results

Morphometric measurements of the trichomes and leaf tissues were carried out in 20 replicates, and the content of the selected elements was determined in four replicates in the three cultivars. The mean values of the measurements and determinations and the standard deviation (stdev. *p*; division by *n*) were calculated with the Microsoft Excel 2013 program. The significance of differences between the cells of the analysed tissues was analysed statistically with the use of Statistica 6.0 software. The differences between the traits were evaluated with one-way analysis of variance (ANOVA). Statistical inference was carried out at the significance level of *p* < 0.05.

## Results

### Morphology of the epidermis

#### Non-glandular trichomes

**Fig. 1:**
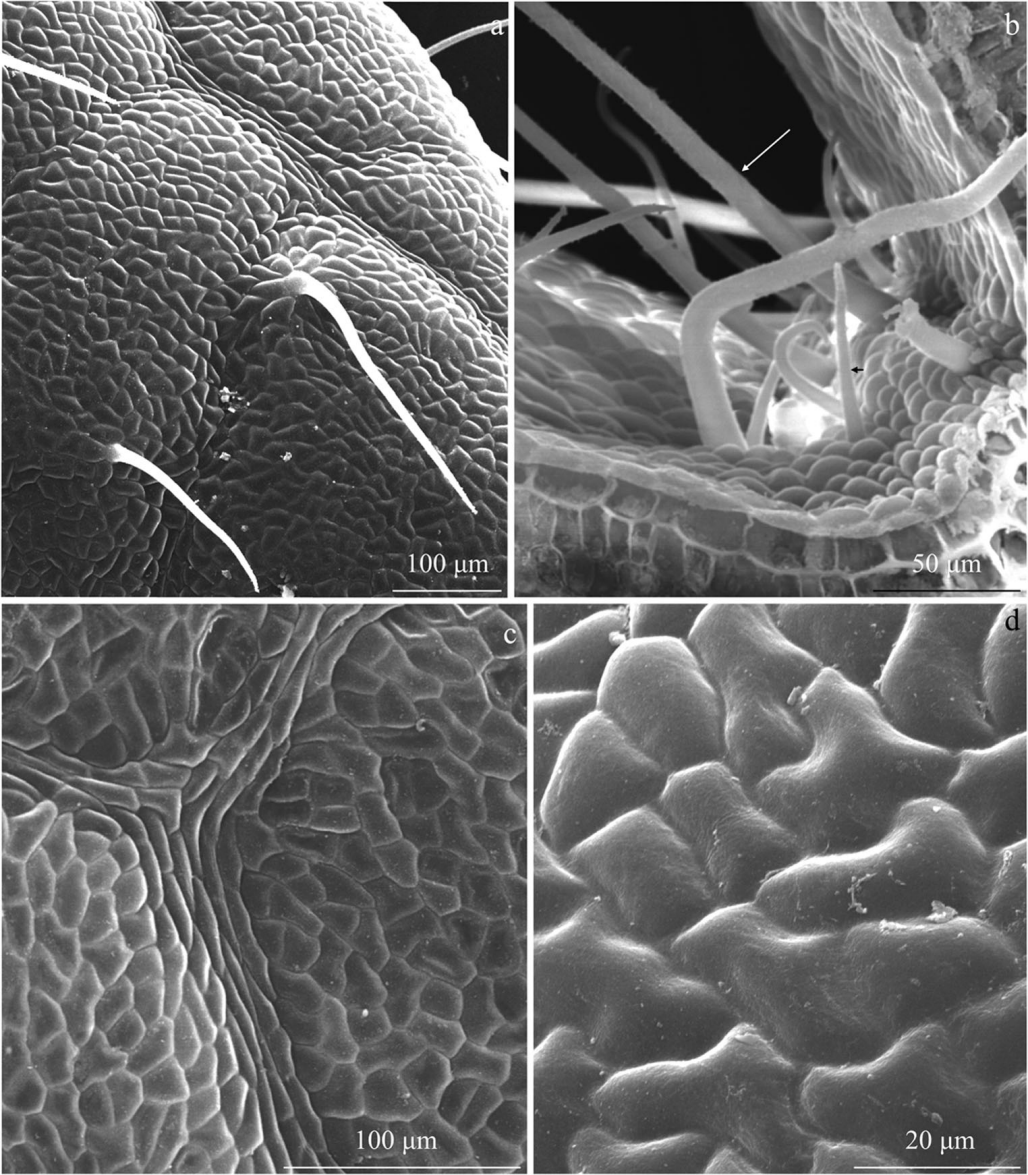
Fragments of the adaxial epidermis surface in the leaves of *R. idaeus* ‘Glen Ample’. **a** Non-glandular trichomes on the epidermis surface and **b** surface of the midrib are visible unicellular non-glandular trichomes varied in length: short (arrowhead) and long (double arrowhead). **c**, **d** Smooth cuticle on the epidermis surface

**Fig. 2:**
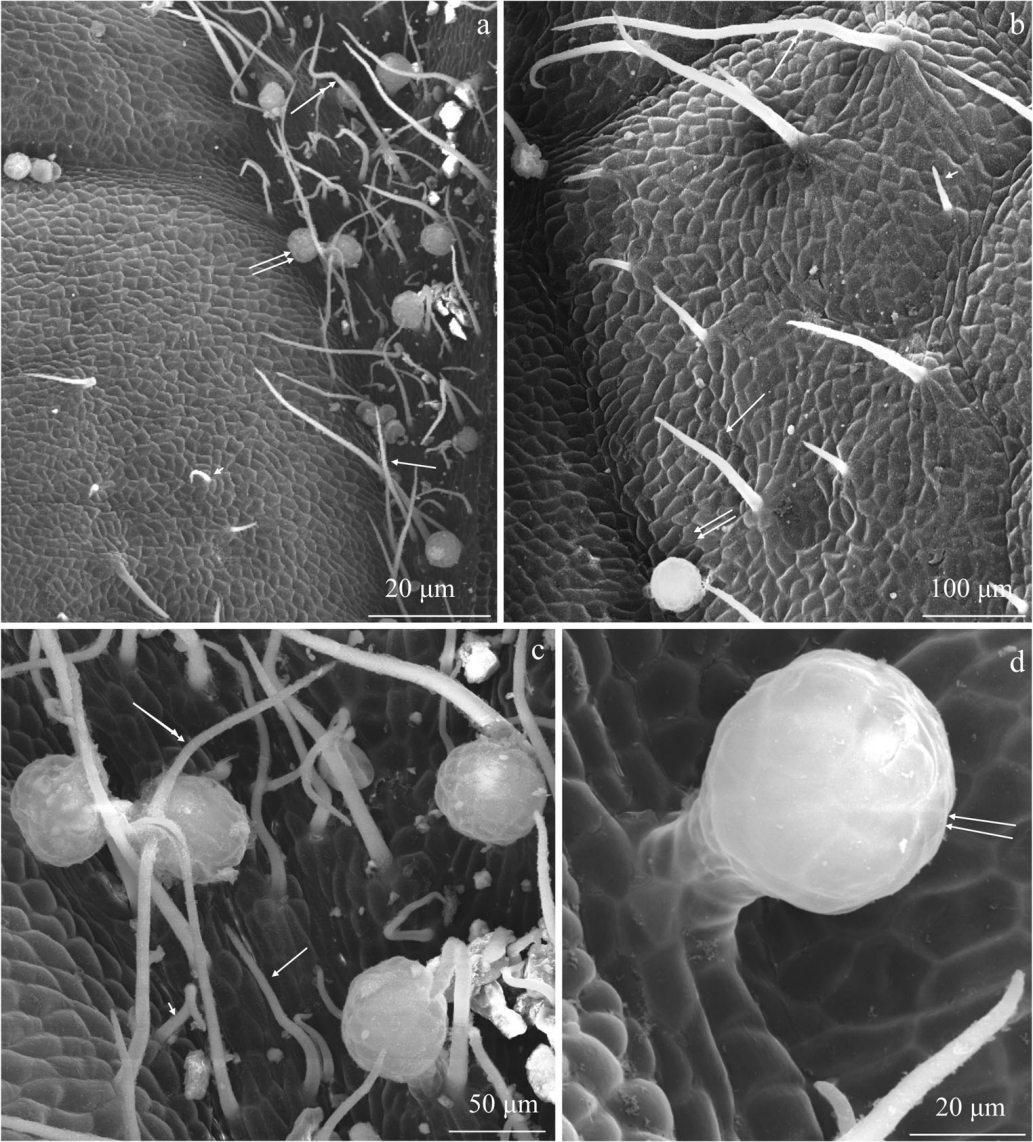
Fragments of the adaxial epidermis surface in the leaves of *R. idaeus* ‘Laszka’. **a**, **b** Non-glandular trichomes: short (arrowhead), medium length (arrow), and long (double arrowhead)—numerous glandular trichomes on the surface of the midrib (double arrows) (**a**) and less dense trichomes on other branching veins (**b**). **c** Glandular trichomes with a multicellular head (double arrows) are visible non-glandular trichomes on the surface of the midrib: short (arrowhead), medium length (arrow), and long (double arrowhead). **d** Glandular trichome with a multicellular, two-layered stalk (in the outline) and a spherical head (double arrows) and smooth cuticle on the epidermis surface

**Fig. 3:**
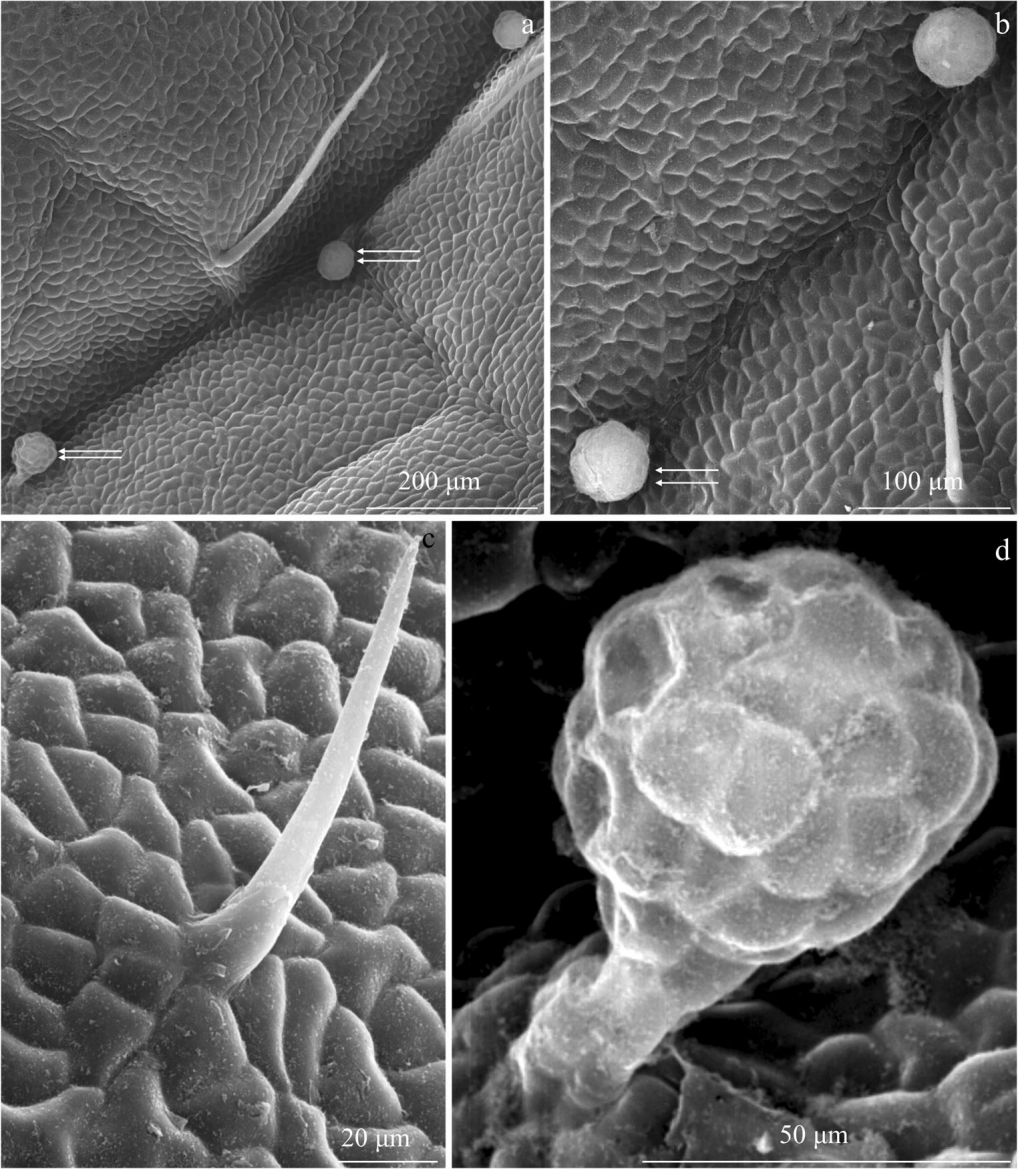
Fragments of the adaxial epidermis surface in the leaves of *R. idaeus* ‘Radziejowa’. **a**, **b** Non-glandular and glandular trichomes (double arrows) growing on the surface of the midrib. **c** Unicellular non-glandular trichome and smooth cuticle with a wax coating. **d** Glandular trichome with a multicellular, two-layered stalk (in the outline) and a multicellular head

There were sparse non-glandular trichomes in the adaxial epidermis of the leaves of the three analysed *R. idaeus* cultivars. The trichomes were unicellular, straight, bristly, and sharply pointed. In terms of the length, short, medium-length, and long trichomes were distinguished (Figs. 1a, b, 2a, b, and 3a–c).

**Fig. 4:**
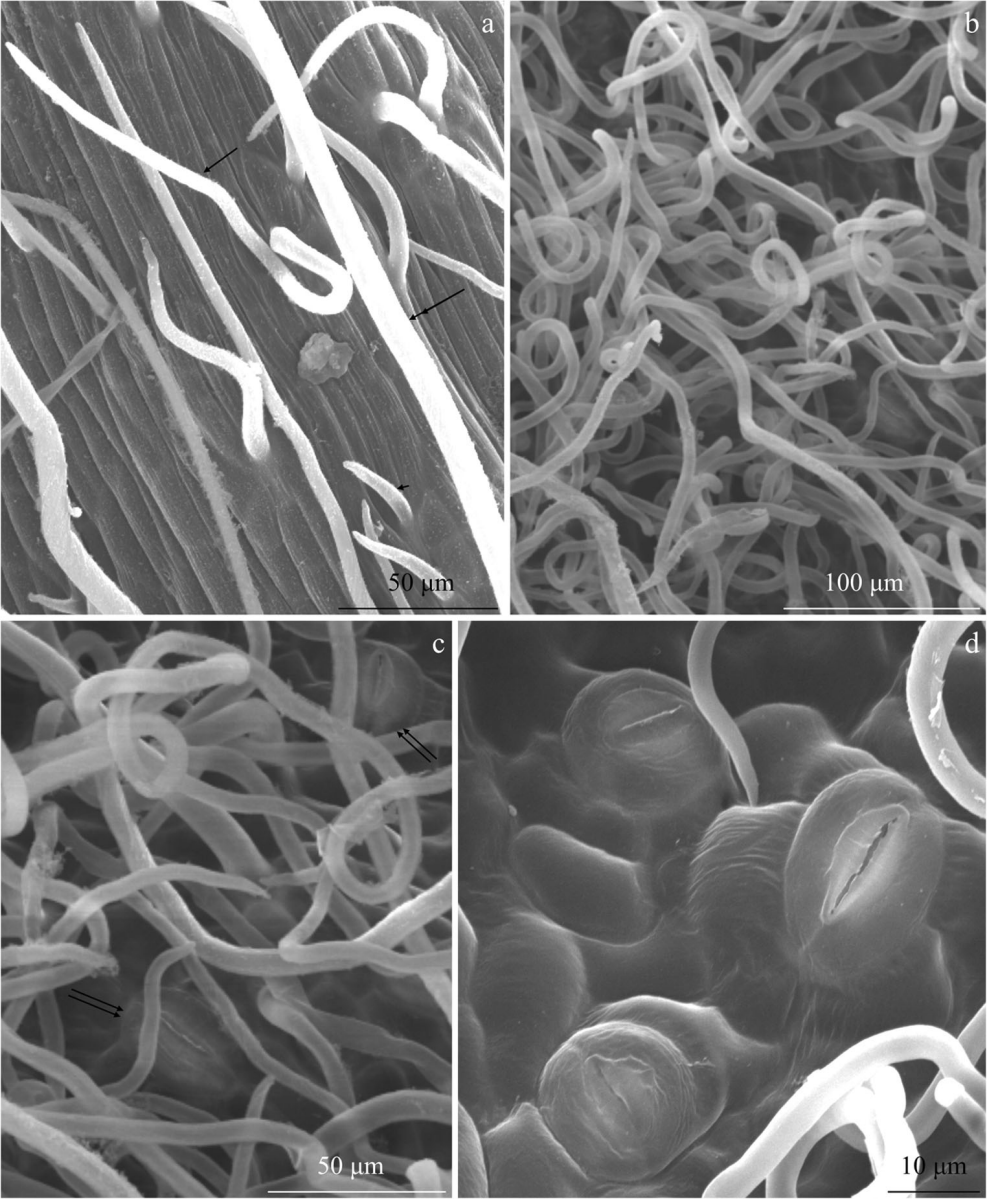
Fragments of the abaxial epidermis surface in the leaves of *R. idaeus* ‘Glen Ample’. **a** Sparse non-glandular trichomes on the surface of the midrib, short (arrowhead), medium length (arrow), and long (double arrowhead), are visible striated cuticle on the surface of epidermis cells. **b**, **c** Densely growing long and twisted non-glandular trichomes and stomata (double arrows) (**c**). **d** Stomata, striated or smooth cuticle on the surface of epidermis cells, and thick cuticular ledges

**Fig. 5:**
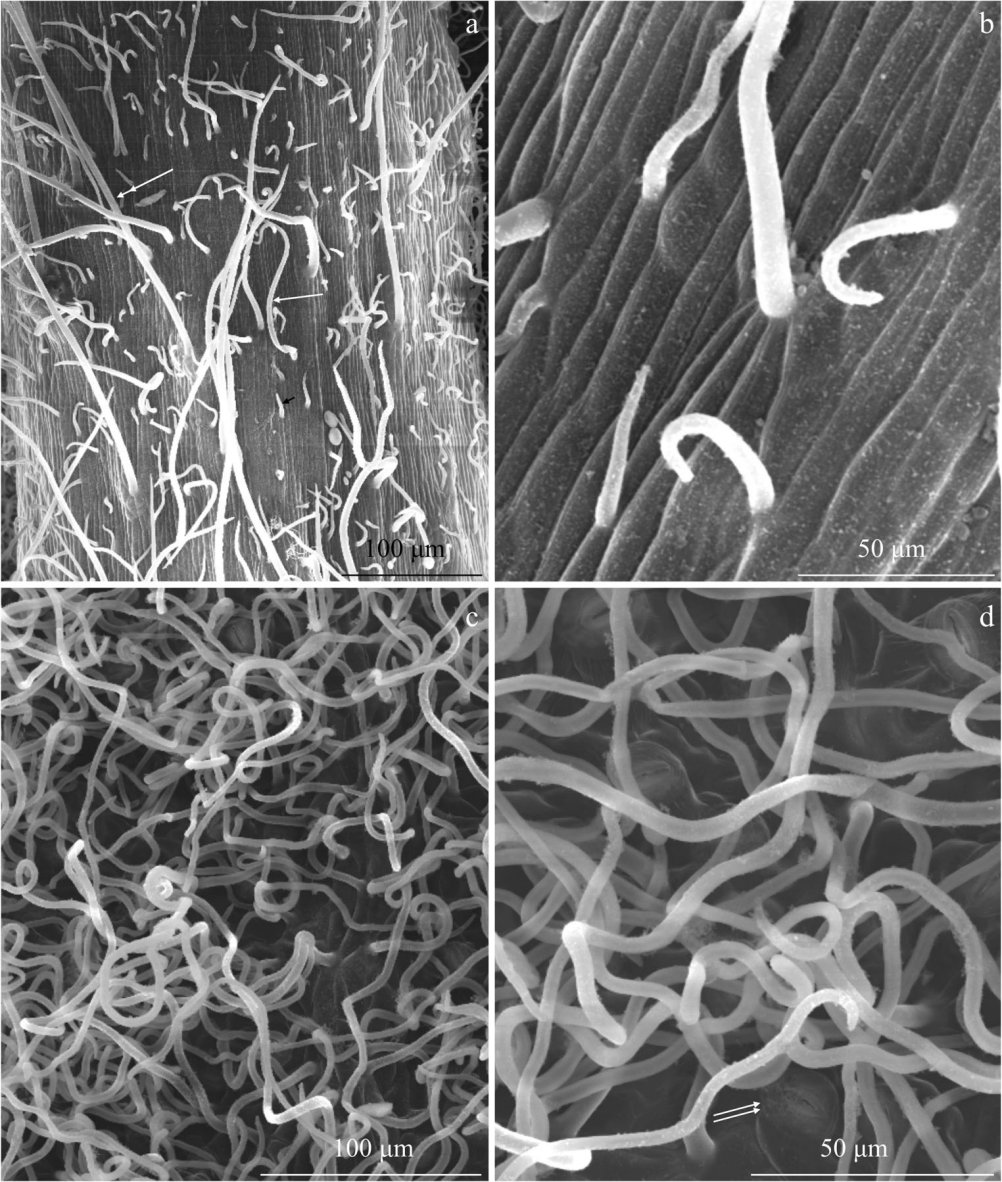
Fragments of the abaxial epidermis surface in the leaves of *R. idaeus* ‘Laszka’. **a**, **b** Trichomes on the surface of the midrib are visible sparse non-glandular trichomes: short (arrowhead), medium length (arrow), and long (double arrowhead) and striated cuticle on the surface of epidermis cells (**b**). **c** Long, twisted non-glandular trichomes masking the epidermis surface. **d** Long, bent non-glandular trichomes and stomata (double arrows) located at the level or above epidermis cells

**Fig. 6:**
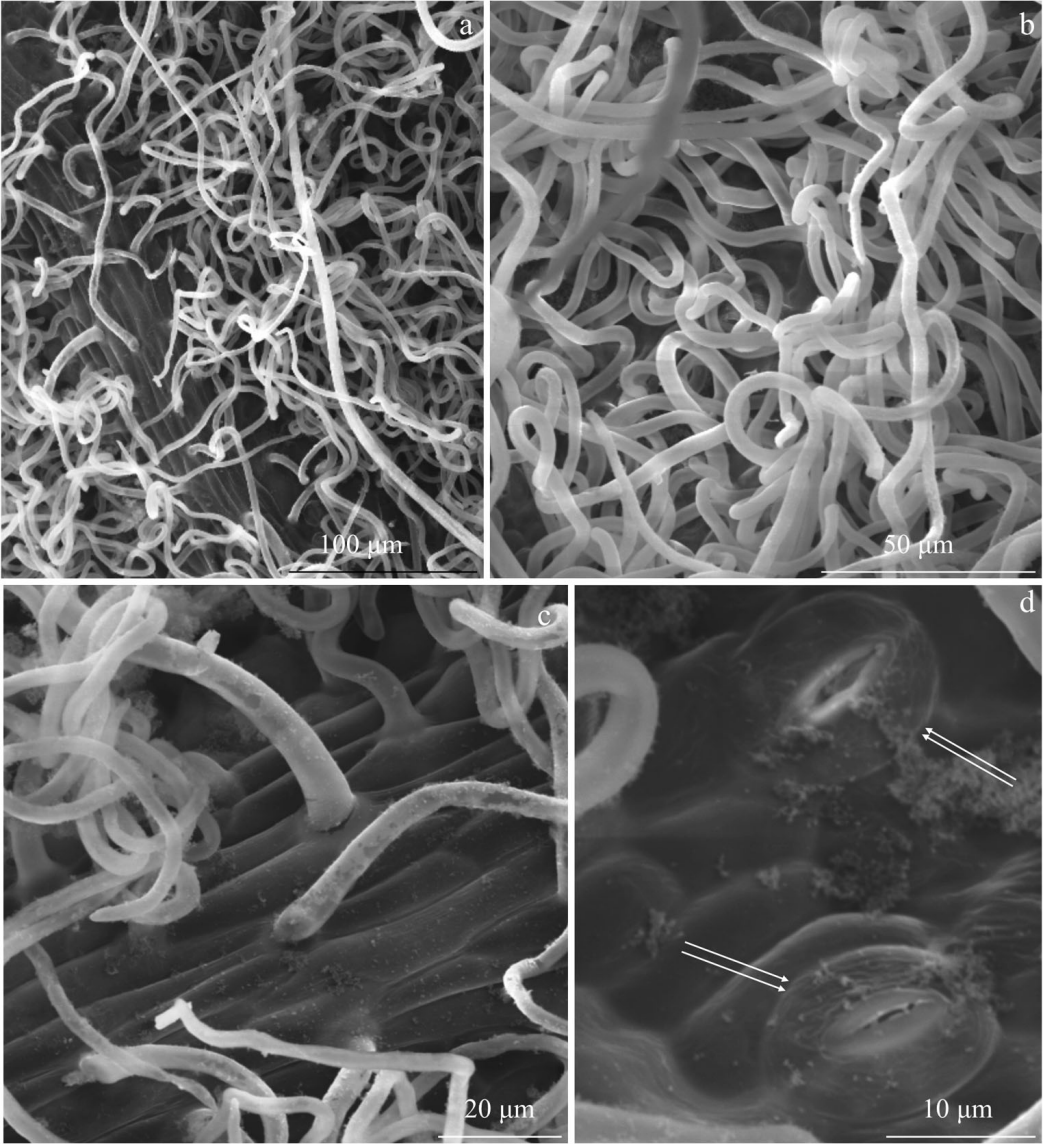
Fragments of the abaxial epidermis surface in the *R. idaeus* ‘Radziejowa’ leaves. **a**, **b** Dense, twisted non-glandular trichomes. **c** Surface of the midrib. **d** Stomata (double arrows), striated cuticle on the surface of stomatal cells, and a smooth or striated cuticle on the surface of other epidermis cells

The trichomes were also present on the surface of the midrib in the abaxial epidermis of the analysed cultivars. In other fragments of the abaxial epidermis, they were denser than those on the midrib surface. The surface of epidermis cells was visible between them (Figs. 4b, 5b, c, and 6c).

The non-glandular trichomes in the intercostal fields in the adaxial leaf epidermis were very long, twisted, and unicellular with a sharply pointed tip. They were distributed densely, masking the epidermis surface (Figs. 4a, b, 5a, c, and 6a, b).

#### Glandular trichomes

**Fig. 7:**
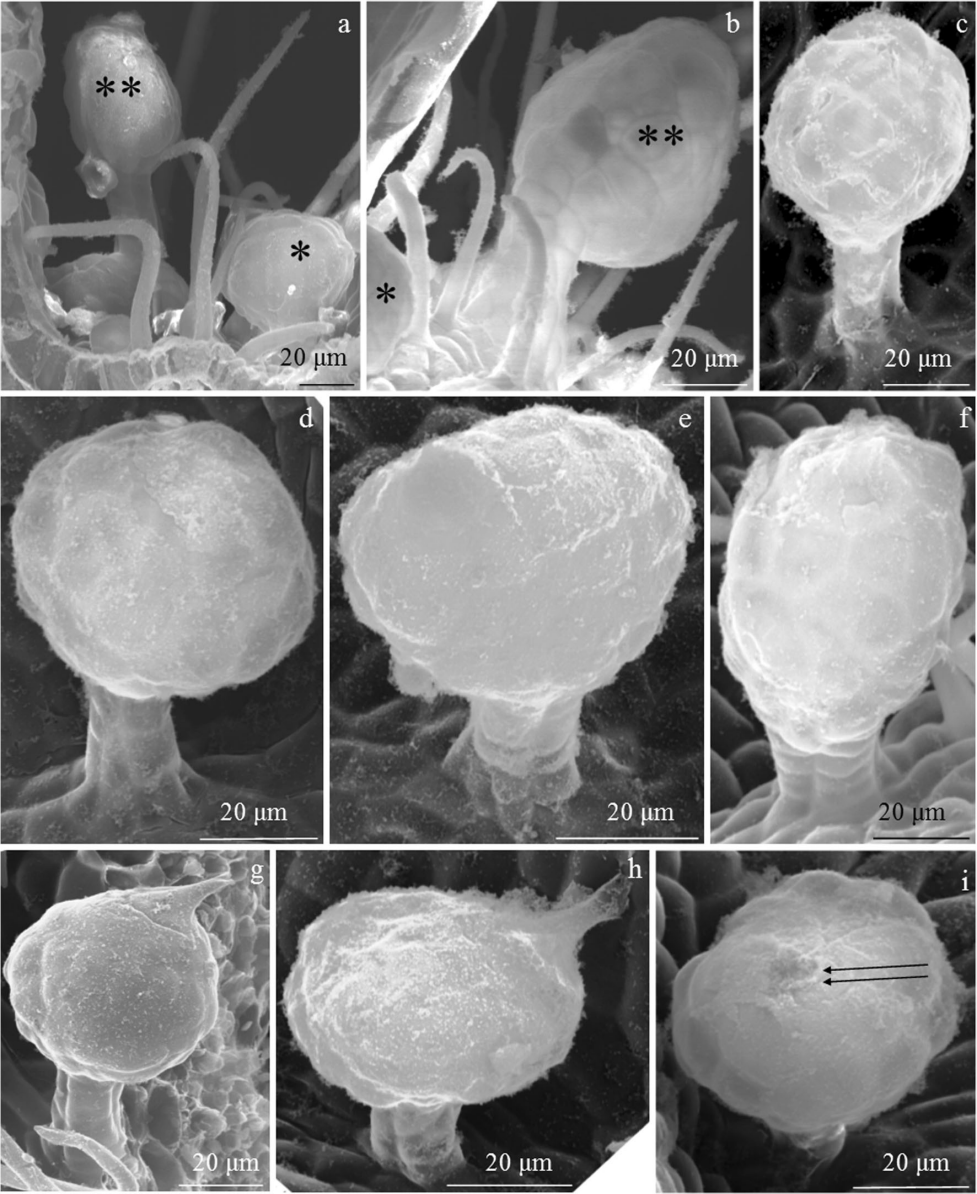
Glandular trichomes in the epidermis of *R. idaeus* ‘Laszka’ and ‘Radziejowa’ leaves. **a**, **b** Visible spherical (asterisk) and elliptical (double asterisk) head of glandular trichomes in the epidermis of the midrib. **c** Glandular trichome with a short stalk and a multicellular head. **d**–**f** Visible two-layered stalk and a spherical (d, e) and elliptical (f) multicellular head of glandular trichomes. **g**, **h** Conical cuticle ruptured at the apex by accumulating secretion. **i** Spherical secretory head with an apical cuticle thickness (double arrows)

Secretory glandular trichomes were observed in the adaxial epidermis of the ‘Laszka’ and ‘Radziejowa’ leaves. No such structures were found in ‘Glen Ample’. The glandular trichomes were located on the surface of the midrib epidermis. They were especially abundant on the midrib in the ‘Laszka’ cultivar (Figs. 3a and 7a) and less dense in ‘Radziejowa’ (Fig. 5a). Glandular trichomes were also observed on successive branches of vascular bundles (Figs. 2b, c and 3a, b).

The glandular trichomes had a multicellular elliptical or spherical head (20–26 cells in the outline) (Fig. 7b–f). Due to exosecretion, the secretion accumulated in the subcuticular space formed a conical protuberance of the cuticle rupturing at the apex (Fig. 7g, h) or a typical spherical bulge (Fig. 7i) at the apex of the trichome head. The height of the secretory head was 42 μm and 53 μm in ‘Radziejowa’ and ‘Laszka’, respectively. Its width was similar, i.e. in the range of 43–45 μm, in both cultivars. The head was located on a multicelled, multiple-layered (2 layers in the outline) stalk. There were 3–7 stalk cells in one layer. The stalk in ‘Radziejowa’ was by 30% longer than the stalk in the ‘Laszka’ epidermis (19 μm). The height of the glandular trichomes in both cultivars was similar (69–71 μm). Their surface in the outline was in the range of 1419–1867 μm^2^ (Table 1).

**Table 1:** Size of glandular trichomes in the adaxial epidermis in two *R. idaeus* cultivars

Trait			Cultivar	
			‘Laszka’	‘Radziejowa’
			Mean ± SD	
Height	Secretory head	μm	42.4 ± 5.4^b^	52.6 ± 3.0^a^
Width			44.5 ± 6.7^a^	43.0 ± 8.1^a^
Surface area		μm^2^	1418.9 ± 182.4^b^	1867.1 ± 269.7^a^
Length	Stalk	μm	27.0 ± 3.7^a^	18.8 ± 2.6^b^
Width			15.0 ± 2.7^a^	14.5 ± 1.9^a^
Surface area		μm^2^	383.9 ± 55.9^a^	270.0 ± 9.1^b^

Means followed by the same letter are not significantly different between cultivars (*p* ≤ 0.05)

#### Histochemistry of glandular trichomes

**Fig. 8:**
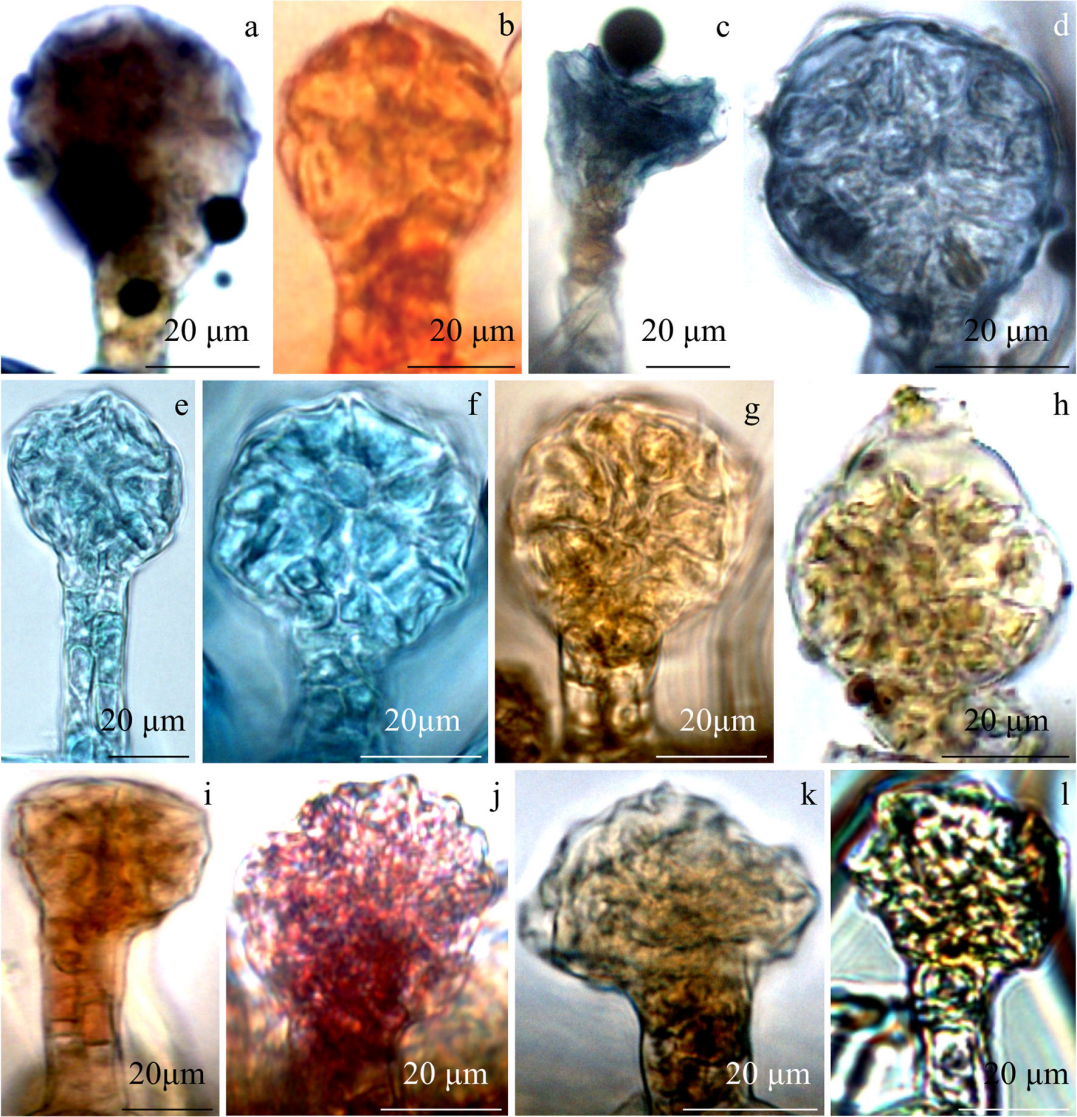
Histochemistry of glandular trichomes in the adaxial epidermis of *R. idaeus* ‘Laszka’ and ‘Radziejowa’ leaves. **a** Terpene compounds stained dark purple (Nadi reagent). **b** Lipid-positive staining reaction (red) (Sudan Red 7B). **c**, **d** Lipid compounds stained dark blue (Sudan Black B). **e**, **f** Acid lipids stained blue (Nile blue). **g**, **h** Pectin compounds stained yellow (Lugol’s solution). **i** Carbohydrates stained brown-red (Fehling’s reagent). **j** Polysaccharides stained pink (periodic acid/Schiff reagent). **k** Polyphenolic compounds stained brown (potassium dichromate) or black (iron chloride)

For the first time, histochemical assays were employed to stain the selected groups of bioactive compounds in the glandular trichomes of *R. idaeus* ‘Laszka’ and ‘Radziejowa’. Various groups of active compounds were detected. In the reaction with the Nadi reagent, terpene compounds present in the glandular trichomes were stained purple (Fig. 8a). The reaction with Sudan Red 7B and Sudan Black B resulted in red (Fig. 8b) and dark blue (Fig. 8c, d) staining of lipid compounds, respectively. Nile blue stained acid lipids blue (Fig. 8e, f). After application of Lugol’s solution, pectin compounds exhibited a positive (yellow) staining reaction (Fig. 8g, h). Fehling’s reagent stained carbohydrates brown-red, whereas PAS reaction stained polysaccharides pink (Fig. 8j). The addition of potassium dichromate or iron chloride yielded brown (Fig. 8k) or black (Fig. 8l) staining of polyphenolic compounds, respectively (Table 2).

**Table 2:** Histochemical tests of the main groups of biologically active compounds in glandular trichomes of the leaves of the three *R. idaeus* varieties

Target compound	Reagent	Colour observed	Glandular trichomes
Nadi reagent	Terpenoids	Purple	+
	Essential oils	Violet-blue	+
Sudan Red	Lipids	Red	+
Sudan Black B	Lipids	Dark blue	+
Nile blue A	Acidic lipids	Blue	+
Lugol’s solution	Proteins	Yellow	+
Fehling’s reagent	Sugars	Brown-red	+
PAS reagent	Polysaccharides	Pink	+
Iron chloride	Phenolic compounds	Brown	+
Potassium dichromate	Phenolic compounds	Black	+

+ indicates positive

#### Cuticle

The outer wall of the adaxial epidermis cells in the analysed *R. idaeus* cultivars was tetra-, penta-, or hexagonal in the outline. The cuticle on its surface was smooth and had a fine wax coating (Figs. 1a, c, d, 2a–d, and 5a–d). In turn, the cuticle was striated on the stomata and on the midrib in the abaxial epidermis and smooth or delicately striated on the other epidermis cells (Figs. 4a, d, 5c, d, and 6c, d).

#### Stomata

The *R. idaeus* leaves are classified as the hypostomatic type. The stomata were located at the level or above the level of the other epidermis cells (Figs. 4d, 5d, and 6d). The length and width of the stomata in the examined cultivars were in the range of 10–22 μm and 8–19 μm, respectively. The largest and the smallest stomata were found in the epidermis of ‘Radziejowa’ and ‘Laszka’, respectively. The stomata were characterised by thick cuticular ledges (1.9–3 μm). The length of the groove between the ledges ranged from 5 (‘Laszka’) to 12 (‘Radziejowa’) μm (Table 3).

**Table 3:** Size of stomata in the abaxial epidermis in the three *R. idaeus* cultivars

Trait (μm)	Cultivar		
	‘Glen Ample’ (2n)	‘Laszka’ (2n)	‘Radziejowa ‘ (2n)
	Mean ± SD		
Length of stomata	17.7 ± 2.6^b^	10.0 ± 1.2^c^	21.8 ± 2.9^a^
Width of stomata	16.2 ± 0.6^b^	7.8 ± 0.6^c^	19.4 ± 1.3^a^
Thickness of cuticular ledge	3.0 ± 0.5^a^	2.7 ± 1.1^b^	1.7 ± 0.3^c^
Length of the groove between cuticular ledges	9.5 ± 1.1^b^	5.3 ± 0.7^c^	11.6 ± 1.4^a^

Means followed by the same letter are not significantly different between cultivars (*p* ≤ 0.05)

### Anatomy

**Fig. 9:**
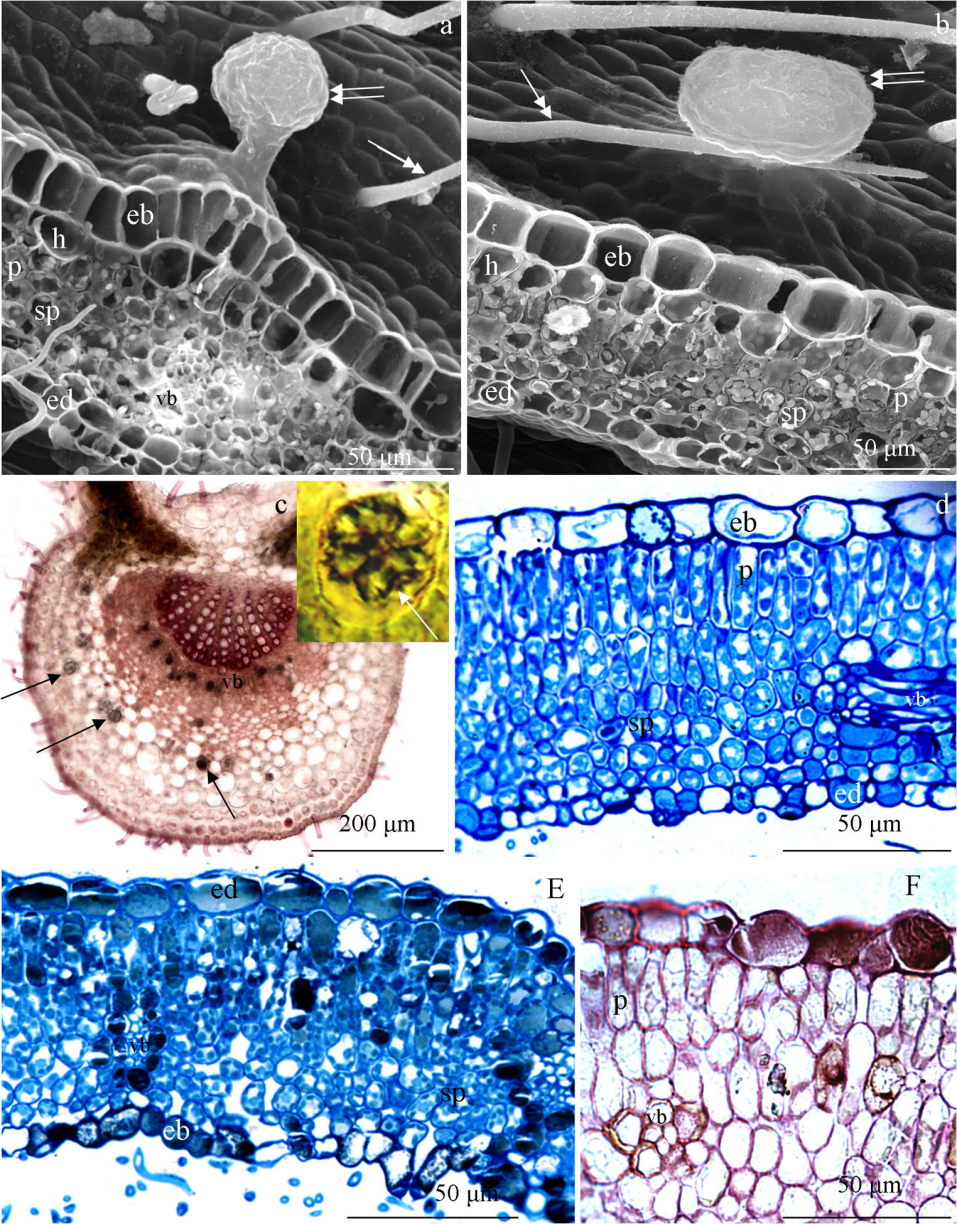
Fragments of cross sections of *R. idaeus* ‘Glen Ample’ (**c**) ‘Laszka’ (**a**, **d**), and ‘Radziejowa’ (**b**, **e**, **f**) leaves. **a**, **b** Non-glandular (double arrowhead) and glandular (double arrows) trichomes, large abaxial epidermis cells (eb) and smaller adaxial epidermis cells (ed), hypodermal cells (h), palisade parenchyma cells (p), and small spongy parenchyma cells (sp) with numerous plastids and vascular bundle elements (vb) (**a**). **c** Midrib and calcium oxalate druses (arrows). **d**–**f** Sections of ‘Glen Ample’, ‘Laszka’, and ‘Radziejowa’ leaves are visible abaxial epidermis cells (eb) and smaller adaxial epidermis cells (ed), one or two layers of palisade parenchyma cells (p), and spongy parenchyma cells (sp) with numerous plastids, vascular bundle (vb), and stomata (s) located above the epidermis cells

The largest and the smallest cells of the adaxial leaf epidermis were observed in ‘Glen Ample’ (19-μm height/24-μm length) and ‘Radziejowa’ (17/17 μm), respectively. An inverse relationship in terms of this trait was found between these cultivars in the abaxial epidermis. The analysed leaves represent the bifacial type (Fig. 9a, b, d–f). The palisade parenchyma cells formed two layers. The largest palisade cells in the subepidermal layer were noted in ‘Glen Ample’ (35-μm height/7-μm width). The values of these parameters in the other two cultivars, i.e. ‘Laszka’ and ‘Radziejowa’, were in the range of 25–29 μm and 8–9 μm, respectively. The thickness of the palisade parenchyma and the lamina in ‘Glen Ample’ and ‘Radziejowa’ was similar, i.e. in the range of 51–52 μm and 121–125 μm, respectively. The thinnest lamina and palisade parenchyma layer were observed in ‘Laszka’. The palisade parenchyma layer in the examined cultivars accounted for approximately 41% of the lamina thickness. The ratio of the palisade to sponge parenchyma in the studied cultivars ranged from 1.1 (‘Laszka’) to 1.3 (‘Glen Ample’) (Table 4). Near the large vascular bands, especially in the main vein, calcium oxalate crystals were visible (Fig. 9c).

**Table 4:** Size of epidermis and mesophyll cells on the leaves of the three *R. idaeus* cultivars

Trait (μm)		‘Glen Ample’	‘Laszka’	‘Radziejowa’
		Mean ± SD		
Height of epidermis cells	Adaxial	19.1 ± 6.6^a^	16.4 ± 3.7^a^	16.8 ± 4.6^a^
	Abaxial	9.4 ± 3.7^a^	9.8 ± 2.3^a^	14.2 ± 4.3^b^
Width of epidermis cells	Adaxial	23.9 ± 6.3^a^	22.1 ± 8.9^a^	16.5 ± 3.0^b^
	Abaxial	9.9 ± 1.7^a^	13.6 ± 3.1^b^	12.4 ± 2.4^b^
Height of palisade parenchyma cells		35.1 ± 3.1^a^	24.6 ± 2.1^c^	28.5 ± 5.0^b^
Width of palisade parenchyma cells		7.0 ± 1.5^c^	7.7 ± 2.1^b^	9.3 ± 2.0^a^
Thickness of parenchyma	Palisade	51.9 ± 7.4^a^	42.1 ± 5.5^b^	51.4 ± 4.7^a^
	Spongy	38.6 ± 7.6^a^	37.9 ± 5.4^a^	40.8 ± 5.5^a^
Thickness of lamina		125.3 ± 5.3^a^	104.7 ± 8.6^b^	121.4 ± 6.4^a^

Means followed by the same letter are not significantly different between cultivars (*p* ≤ 0.05)

### Ultrastructure of epidermal and assimilation cells

**Fig. 10:**
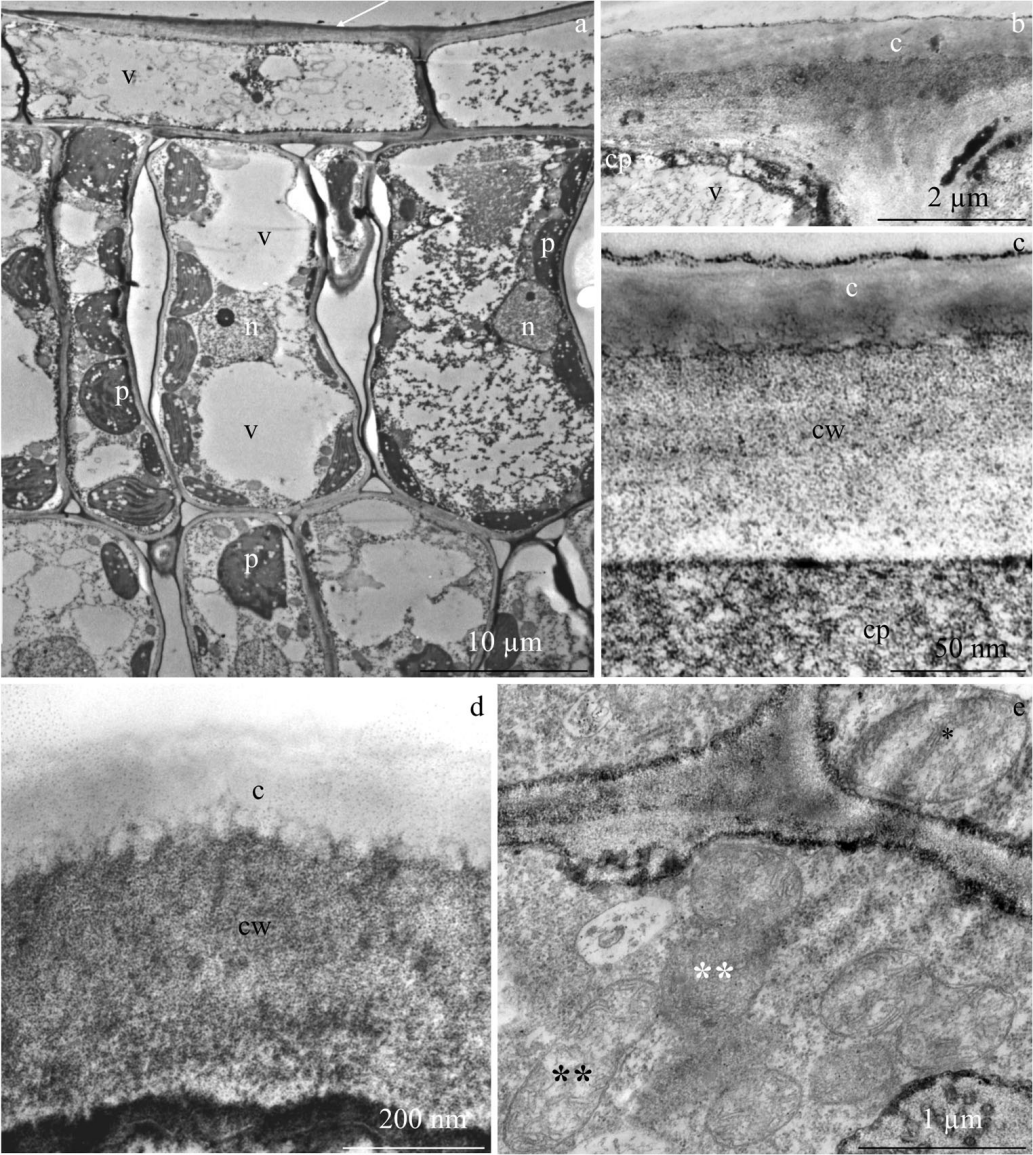
Fragments of cross sections of the cells of adaxial epidermis and assimilation parenchyma in *R. idaeus* ‘Radziejowa’ leaves. **a** Adaxial epidermis and palisade mesophyll cells, thick cell walls (arrow) and a large central vacuole (v) in the epidermis cells, numerous plastids (p), large cell nucleus (n) with dense nucleoplasm, nucleolus, and varied degree of vacuolization in assimilation cells. **b** Epidermis cells, periclinal outer cell wall (cw), cytoplasm, and vacuole (v). **c**, **d** Epidermis cells, cuticle strand (**c**), the other part of the cell wall (cw), and cytoplasm. **d** Single mitochondria in the epidermis protoplast (asterisk) and mitochondria clustered in the assimilation cell (double asterisk)

**Fig. 11:**
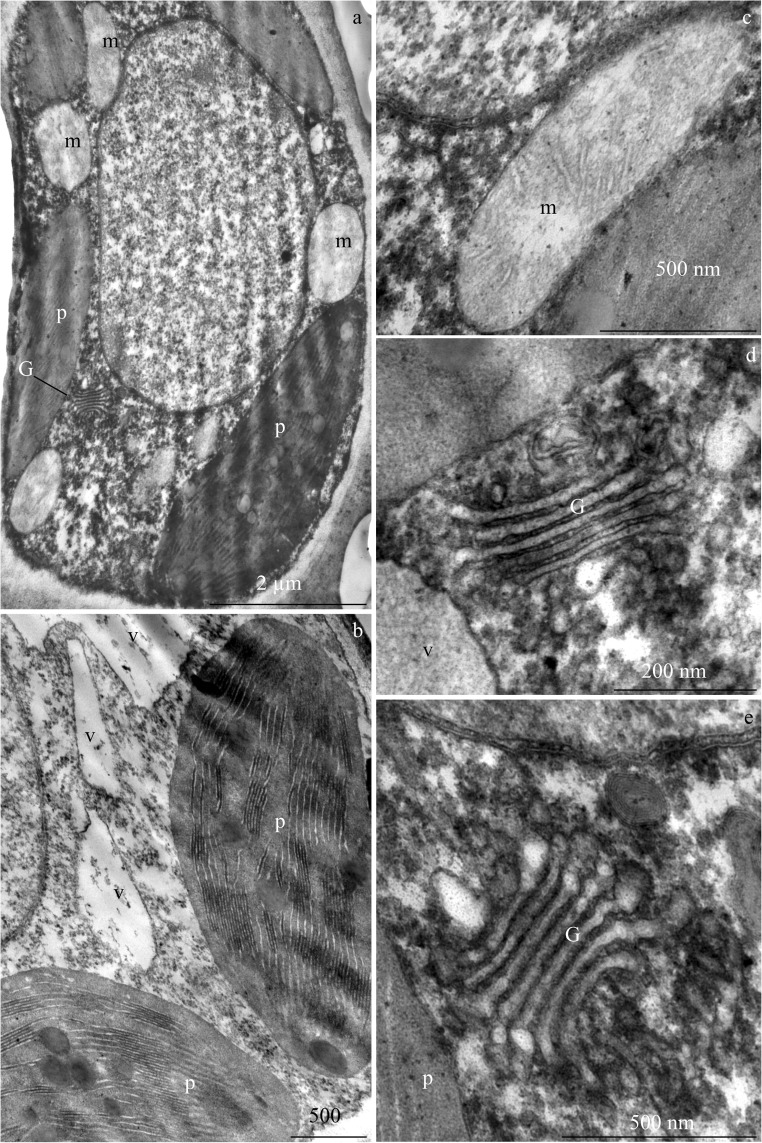
Fragments of the cross sections of palisade mesophyll cells in *R. idaeus* ‘Radziejowa’ leaves. **a** Protoplast with parietal cytoplasm, which is a visible plastid (p), mitochondria (m), and Golgi apparatus. **b**, **c** Plastids (p) with a thylakoid system. **c** Mitochondrion. **d**, **e** Golgi apparatus (G) with cisternae and dictiosomal vesicles

The epidermis cells in *R. idaeus* leaves produced a thick periclinal outer cell wall (Fig. 10a). On the surface of the epidermis, there was a cuticle strand (Fig. 10b–d). The protoplast of these cells contained a parietal cytoplasm and single mitochondria with a well-developed membrane system (Fig. 10e). The palisade mesophyll cells had a large nucleus with a thick nucleoplasm and a dark nucleolus, varied degrees of vacuolization (Fig. 10a), numerous plastids with a distinct thylakoid system (Figs. 4b and 11a), mitochondria (Fig. 11a, c), and Golgi apparatus composed of 4–6 cisternae and a number of dictiosomal vesicles (Fig. 11d, e).

### Microanalysis of elements in selected parts of epidermis cells

**Fig. 12:**
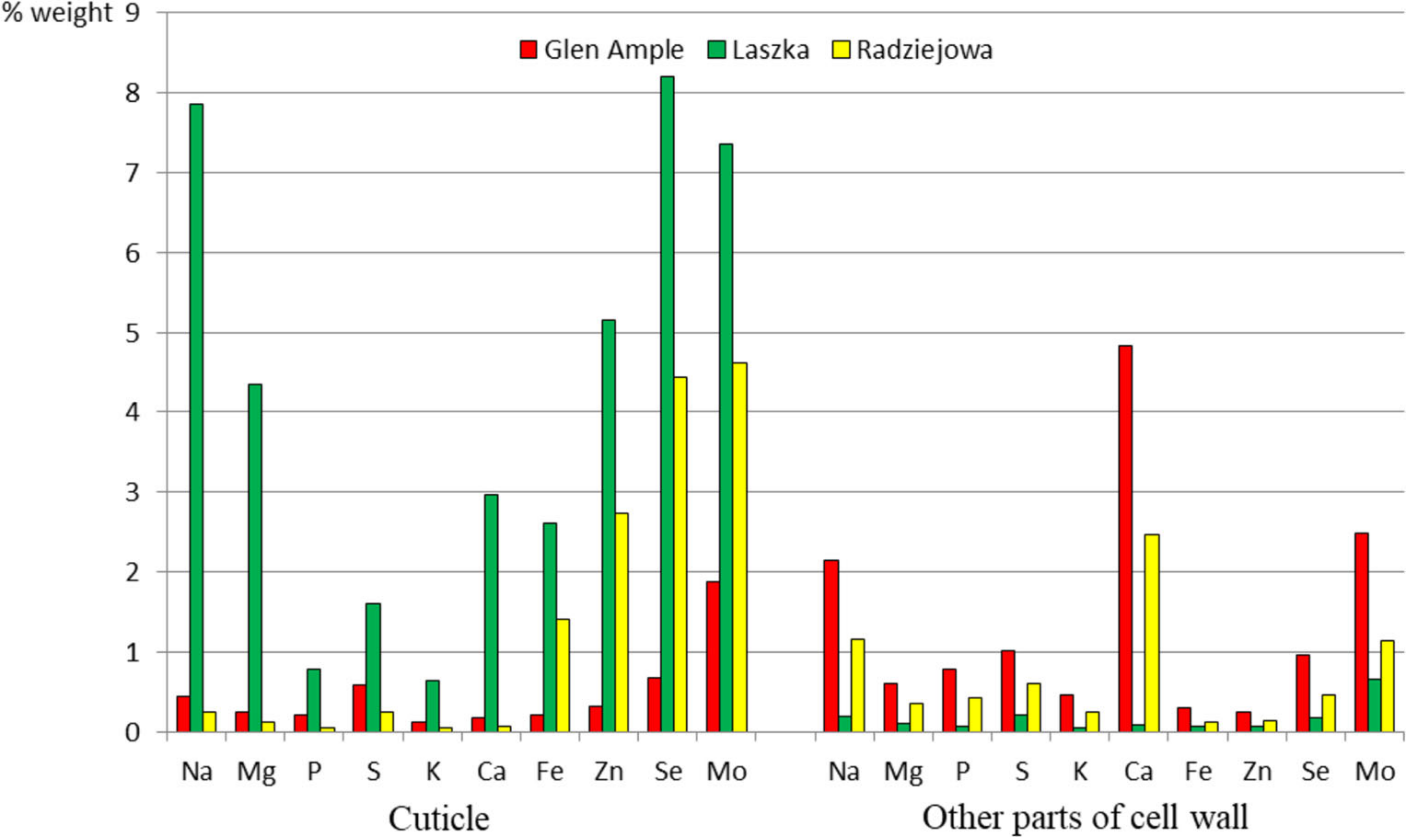
Content of selected elements in the cuticle and other parts of the cell wall of epidermal cells in the leaves of the three *R. idaeus* cultivars

**Fig. 13:**
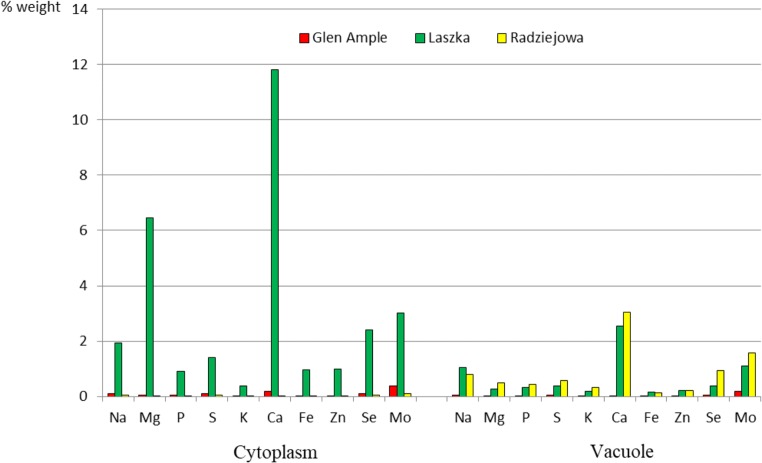
Content of selected elements in the cytoplasm and vacuole of epidermal cells in the leaves of the three *R. idaeus* cultivars

The microanalysis of the selected elements at the level of the ultrastructure of epidermis cells (cuticle, the rest of the cell wall, cytoplasm, and vacuoles) revealed their highest concentrations in ‘Laszka’. The cuticle layer was predominated by Na, Se, and Mo. In turn, the other part of the cell wall exhibited the highest concentrations of Ca, Na, and Mo in ‘Glen Ample’ and ‘Radziejowa’. In turn, there was a substantial concentration of Ca, Mg, and Mo in the cytoplasm of ‘Laszkaʼ. Ca, Mo, Na, and Se dominated in ‘Laszka’ and ‘Radziejowa’ (Figs. 12 and 13).

### Content of elements in leaf dry mass

The concentration of the K, Ca, Mg, P, and Fe elements in the analysed *R. idaeus* leaves differed between the cultivars. The elements were aligned according to the growing content as follows: K > Ca > Mg > P > Fe (‘Glen Ample’), K > Ca > P > Mg > Fe (‘Laszka’), and K > Mg > Ca > P > Fe (‘Radziejowa’). The amount of sodium in the three examined cultivars was below the limit of determination. In turn, the content of zinc was similar in the leaves of ‘Laszka’ and ‘Radziejowa’ (40 mg/kg d.w.), and by ca. 14% lower in ‘Glen Ample’ than in the other two cultivars. The highest concentration of copper was found in ‘Laszka’ (34.1 mg/kg d.w.), while the other two cultivars were characterised by a ca. 68% lower concentration of this element. The zinc content in the leaves of the three cultivars was similar and ranged from 34 to 40 mg/kg d.w. Trace elements, i.e. cadmium and lead, were present at very low concentrations (well below the permissible levels) in the leaves of the analysed cultivars (Table 5).

**Table 5:** The content of elements in the leaves of the three varieties of *R. idaeus*

Element content	‘Glen Ample’	‘Laszka’	‘Radziejowa’
	Mean ± SD (mg/kg)		
Potassium	12,600 ± 100^c^	20,000 ± 298^a^	14,100 ± 203^b^
Calcium	4800 ± 159^a^	2730 ± 155^b^	2610 ± 82^b^
Magnesium	2800 ± 109^b^	2100 ± 115^c^	3610 ± 121^a^
Phosphorus	2550 ± 239^a^	2463 ± 190^ab^	2090 ± 146^b^
Iron	176 ± 16^c^	208 ± 18^b^	252 ± 19^a^
Sodium	< 0.250^a^	< 0.250^a^	< 0.250^a^
Copper	12 ± 1^b^	34 ± 1^a^	11 ± 0.4^b^
Zinc	34 ± 3^a^	40 ± 3^a^	40 ± 3^a^
Lead	0.0018 ± 0.0001^b^	0.0021 ± 0.0003^ab^	0.0023 ± 0.0002^a^
Cadmium	0.0026 ± 0.0003^a^	0.0029 ± 0.0003^a^	0.0007 ± 0.0001^b^

Means followed by the same letter are not significantly different between cultivars (*p* ≤ 0.05)

## Discussion

### Micromorphology

The hypostomatic type of leaf lamina of the *R. idaeus* cultivars analysed in this study is present in *Prunus laurocerasus* (Schreiber et al. 1995) as well as plants from the genera *Sorbus* and *Sorbaria* (Krivoruchko and Gamulya 2013; Song and Hong 2014). As shown by our investigations and literature data, the abaxial epidermis in *R. idaeus* had twisted unicellular non-glandular trichomes (Fell and Rowson 1960; Tomaszewski et al. 2014; Karley et al. 2015). Their length in the epidermis of *R. loganobaccus* leaves ranged from 0.3 to 0.7 mm (Fell and Rowson 1960). The non-glandular trichomes in the three cultivars analysed in the present study can be classified according to Tomaszewski et al. (2014) as the simple unbranched type and three different subtypes (short, medium-length, and long). According to the classification of trichomes proposed by Payne (1978), acerate, aduncate, angler, arrect, and attenuate protective trichomes were distinguished in the epidermis of *R. idaeus* leaves.

The non-glandular trichomes in the intercostal fields in the abaxial epidermis were densely distributed, masking the epidermis surface in the three *R. idaeus* cultivars. As reported by Upadhyaya and Furness (1998), there were 7–82 trichomes per 1 mm^2^ of the epidermis in several *R. idaeus* cultivars. According to the division proposed by Tomaszewski et al. (2014), based on the density of non-glandular trichomes, the non-glandular trichomes in the adaxial epidermis described in the present study can be classified into the first group (< 10%), while those present in the abaxial epidermis represent the third or fourth group (> 90%).

The size and topography of trichomes are modified by environmental conditions (Upadhyaya and Furness 1998; Wilkens et al. 1996). Non-glandular trichomes in the epidermis serve various functions. They constitute a protective barrier against pests, e.g. from the genera *Amphorophora* and *Tetranychus* (Levin 1973). The presence of trichomes on the epidermis surface was found to reduce the number of pests feeding on *R. idaeus* leaves (Karley et al. 2015). Non-glandular trichomes are involved in adaptation to adverse environmental conditions. They produce hydrophilic substances that help to retain water, thereby preventing drying out and promoting the growth of young organs. This hyaline material contained mainly carbohydrates derived from the partial degradation of the trichome cell wall (Lusa et al. 2015). The secretion of colleters on the surface of buds and floral elements protects against water loss as well (Mayer et al. 2013). Some glandular trichomes provide food for pollinators (Pansarin and Maciel 2017), while others secrete terpenoids, phenolic compounds, and insect-repellent proteins (Lusa et al. 2015). Accumulation of e.g. terpenoids in trichomes suggests a chemo-ecological function in the interaction of plants with insects or herbivores (Amrehn et al. 2014).

A similar structure of glandular trichomes to that shown in this study, i.e. the presence of a multicellular or spherical head located on a multicelled multiple-layered (2 layers in the outline) stalk, has been described in the leaf epidermis of several *Rubus* species (Fell and Rowson 1961). Glandular trichomes produce bioactive substances with various properties for pharmaceutical and industrial applications (Kjær et al. 2012; Tozin et al. 2017; Chwil and Kostryco 2018; Chwil et al. 2013; Mayer et al. 2013; Weryszko-Chmielewska and Chwil 2017). Excretion from a periplasmic space of the upper cell layers pass through the cell wall, accumulate in the subcuticular cavity, and rupture it (Muravnik et al. 2019). Depending on the function, each morphotype of these glands is characterised by varied responses in the defence of plants against environmental factors (Tozin et al. 2017; De Vargas et al. 2019). It has been found that the characteristics of trichomes and stomata are associated with immunity of mulberry to powdery mildew and can be indicators of this type of immunity in cultivation programs (Chattopadhyay et al. 2011).

A stable trait of trichomes in some *R. idaeus* cultivars was used in genetic engineering to develop new varieties (Upadhyaya and Furness 1998). The cultivation work resulted in identification of chromosomal regions of genes encoding and regulating micromorphological traits that can be genetically controlled (Graham et al. 2014). Therefore, some species with favourable traits, e.g. *R. canescens*, are often used in the development of new taxa (Tomaszewski et al. 2014).

Kellogg et al. (2011) suggest that the glandular trichomes of *R. idaeus* give rise to prickle primordia, which transform into thorns. A proportional relationship was found between the size of the trichome heads and the size of the developing thorns. In the present study, thorns were produced by the ‘Laszka’ and ‘Radziejowa’ cultivars, which had glandular trichomes, whereas the thornless ‘Glen Ample’ cultivar had no glandular trichomes. The presence of mechanical and glandular trichomes on the surface of the leaf epidermis was detected in many species from the genus *Rubus* (Fell and Rowson 1961).

### Histochemistry

The histochemical assays helped to identify various groups of biologically active compounds. The Nadi reagent stained terpene compounds purple. A similar colour of terpenes was also detected in *Rosa* × *damascena* trichomes (Caissard et al. 2005). This group of compounds in *Rubus suavissimus* was represented by the dominant rubusoside and steviol monoside (Chou et al. 2009). Another chemotaxonomic function is served by diterpene glycosides identified in *R. chingii* leaves (Chou et al. 1987).

Lipid compounds in the trichomes were stained red, dark blue, or light blue using Sudan Red 7B, Sudan Black B, or Nile blue, respectively. The compounds were characteristically stained in the trichome secretory head and stalk.

A positive result of histochemical assays of the presence of lipids in glandular trichomes was reported in the case of *Melissa officinalis* (Chwil et al., 2016). In our previous study of *Rubi folium* from ‘Laszka’, ‘Radziejowa’, and ‘Glen Ampleʼ, the fatty acids were dominated by palmitic, arachidonic, tetracosanoic, and stearic acids. The main omega 3, omega 6, and omega 9 acids were α-linolenic acid, linolenic acid, and oleic acid, respectively (Chwil and Kostryco 2018).

A substantial proportion of fat is contained in epicuticular wax. These are mainly long-chain aliphatic compounds and terpenyl esters (α- and β-amyrin and cycloartenol). By forming the structure of wax, these compounds provide protection against some organisms (Shepherd et al. 1999). A similar role of metabolic resistance to pathogens is played by the proteins identified in *R. idaeus* leaves (Colditz et al. 2007; Hukkanen et al. 2008).

A positive (yellow) staining reaction of protein compounds in the stalk and the secretory head was exhibited after application of Lugol’s solution. On average, this group of compounds accounted for 20% of air-dried matter of *R. idaeus* leaves (Chwil and Kostryco 2018). Proteins exhibit developmental dependence (De Nardi et al. 2006). They can bind hydrophobic ligands (flavonoids and plant hormones) (Fernandes et al. 2008; Mogensen et al. 2007) and serve as flavonoid-type transporters (Hjernø et al. 2006).

Carbohydrates were stained brown-red with Fehling’s reagent, whereas the PAS reaction stained polysaccharides pink. These compounds yielded a positive staining reaction in the glandular trichomes in *M. officinalis* (Chwil et al. 2016). The average content of absorbable carbohydrates and sugars in the leaves of the analysed species was 6% and 5%, respectively, as reported in the literature. In turn, total fibres constitute 58% with 4 g/100 g of the soluble fraction and 54 g/100 g of the insoluble fraction (Chwil and Kostryco 2018). Extracts of raw *R. suavissimus* leaves were reported to contain 11% of polysaccharides (Koh et al. 2009). Sugars detected in *R. chingii* were represented by rhamnose, arabinose, xylose, glucose, and galactose. These bioactive compounds isolated from the leaves of this species exhibited antioxidant, anti-inflammatory, and anticancer activity (MCF-7 and Bel-7402). Given their bioactivity, these compounds can be a source of food additives (Zhang et al. 2015).

As in other literature reports, polyphenolic compounds analysed in the present study were stained black or brown with potassium dichromate or iron chloride respectively (Jachuła et al. 2018). In various *Rubus* species, these compounds have been reported to exhibit antioxidant capacity determined with in vivo and in vitro methods (Dall’Acqua et al. 2008; Chwil and Kostryco 2018). The antioxidant group was dominated by caffeic acid, ferulic acid, quercetin, kaempferol, chlorogenic acid, caffeoylquinic acid (Dall’Acqua et al. 2008). The highest total antioxidant activity in the leaves of the analysed *R. idaeus* cultivars determined with the FRAP and Folin-Ciocalteu methods was found for ‘Radziejowa’ (Chwil and Kostryco 2018). The concentration of phenolic compounds in the leaf extracts from the different *Rubus* species was significantly correlated with the antioxidant activity (Oszmiański et al. 2015; Om et al. 2016). Szymanowska et al. (2018) reported antioxidant and anti-inflammatory properties of phenolic compounds and anthocyanins contained in *R. idaeus* fruit extracts. Their anti-inflammatory action was manifested by an inhibitory effect on the activity of lipoxygenase and cyclooxygenase-2 in vitro. These extracts effectively inhibited the viability of human leukaemia cells J45 and HL60 in in vitro investigations of cytotoxic activity. As demonstrated by Manríquez-Torres et al. (2016), fruits of various *Rubus* species exhibited high antioxidant activity due to their content of anthocyanins and other antioxidants.

### Anatomy

The epidermis of the examined leaves was formed of large cells with a thick outer wall and a cuticle layer. The palisade parenchyma cells formed two layers. A similar number of palisade parenchyma layers were found in the leaves of *R. idaeus* and *Prunus serotina* (Fell and Rowson 1961; Ferdinand et al. 2000), wherein *R. idaeus* cultivars had a more pronounced double palisade parenchyma than the wild taxa. The size of the palisade parenchyma cells in the present studies is in the range of values specified for *R. idaeus* in literature reports (Fell and Rowson 1960). These traits are ecological adaptations to environmental conditions allowing more efficient utilization of solar radiation (Kołodziejek et al. 2015).

The distribution of mesophyll cells in these leaves and leaves of plants from the genera *Sorbus* and *Pyrus* described in the literature indicated a bifacial type (Zamani et al. 2008; Krivoruchko and Gamulya 2013). A well-developed palisade parenchyma determines efficient photosynthesis and is an ecological strategy of adaptation to environmental conditions. This parenchyma utilises solar light more efficiently than the spongy tissue. Plants from insolated habitats are characterised by a well-developed palisade parenchyma. In turn, the amount of the spongy parenchyma increases in shaded environments (Kołodziejek et al. 2015). The anatomical structure exhibited the presence of numerous calcium oxalate crystals located in a particularly large number close to a large vascular bundle in the leaf midrib. These precipitates have been described in leaves of several species from the genus *Rubus* (Fell and Rowson 1960, 1961).

### Content of elements

The content of elements in raspberry leaves largely depends on the mode of cultivation and the environment. Potassium and calcium followed by magnesium and phosphorus dominated in the leaves of the analysed raspberry cultivars. This is evidenced by the large variation of the quantities of these elements in the leaves of raspberry cultivated in different remote regions of Lublin Province, south-eastern Poland (Dresler et al. 2015). The results of the K, Mg, and P content presented in this study are within the ranges specified by the aforementioned authors for these elements, i.e. K (10,600–20,500 mg/kg), Mg (2600–4500 mg/kg), and P (1900–3000 mg/kg), in *R. idaeus* leaves. In turn, the concentration of Ca (2610–4800 mg/kg) determined in this study is lower than the content of this element (7200–15500 mg/kg) in the raw material collected by these researchers. These mineral elements are components of enzymes and proteins as well as important elements of biochemical processes and tissue structures in the human organism (Higdon 2003).

Assessment of the nutritional value in terms of the content of elements in the leaves of the raspberry cultivars analysed in this study can be based on comparison of their concentrations in the leaves of different *Camellia sinensis* cultivars. The Ca, Mg, Cu, Fe, and Zn content reported in the present study was similar or higher, the amount of K and P was in the same range, and the level of Na, Pb, and Cd was lower as compared with that in green tea leaves. In turn, the Fe content in the examined *R. idaeus* cultivars was substantially higher than the concentration of this element in *Camellia sinensis* leaves (Chen et al. 2009). Milošević et al. (2018) reported that blackberry fertilization with NPK, manure, natural zeolite, and their mixtures changed a majority of cane and berry physical traits and leaf nutrient status. Increased meso-nutrients improve the growth of micropropagated red raspberries. The ratio of ammonium to nitrate varies greatly for improving plant growth. An important step is to determine the driving mineral factors in improved medium formulations for micropropagated red raspberries (Poothong and Reed 2014).

The content of Ca in our studies were from half to three-fold lower than that in leaf blades in *R. coreanus.* In turn, the content of K was lower in ‘Glen Ample’, equal in ‘Radziejowa’, and greater in ‘Laszka’ than in *R. coreanus.* In leaves ‘Glen Ample’ and ‘Radziejowa’, the higher Mg and Cu as well as lower content than that in *R. coreanus* was found (Om et al. 2016).

The content of trace elements, i.e. cadmium (0.0007–0.0029 mg/kg) and lead (0.0018–0.0023), in the examined *R. idaeus* leaves was significantly lower than the permissible levels specified for plant raw materials (Wang et al. 2015). Lead and cadmium are very harmful to humans (Grytsyuk et al. 2006). Lead is toxic to the circulatory, nervous, and digestive systems and accumulates in bones (Glenn et al. 2001; Gidlow 2004). In turn, cadmium damages kidneys, bones, and the respiratory system (Godt et al. 2006). The content of Cd and Pb in the leaves of the *R. idaeus* cultivars analysed in the present study and described in the literature (Cd 0.0026 mg/kg, Pb 0.0018–0.0023 mg/kg) is substantially lower than that in *Camellia sinensis* leaves (Cd 0.05 mg/kg, Pb 2.28–4.33 mg/kg) (Chen et al. 2009). This indicates that the raspberry shrubs analysed in this study grow on unpolluted and uncontaminated soil in the clean environment of the Lublin Upland located in the east-south of Poland.

## Conclusions

The knowledge of the micromorphology and anatomy of glandular trichomes, which are present in the vegetative parts of plants from the family Rosaceae, is highly important in the verification of the authenticity of herbal raw material. The present study provides information in the field of plant biology, more specifically botany, regarding glandular trichomes as a site of accumulation of secondary metabolites used e.g. in pharmaceutical, food, cosmetics, and chemical industries. These phytochemicals play a key role in plant communication with the environment, including plant-animal interactions, the ecology of pollination, and plant protection against unfavourable environmental factors. This important scientific information can be used in agricultural and horticultural practice by herbalists, dieticians, pharmacists, cosmetologists, and beauticians.

The glandular trichomes with a multicellular head and a multilayer stalk were present on the epidermis in ‘Laszka’ and ‘Radziejowa’. The micromorphology of the abaxial *Rubus idaeus* epidermis exhibited a striated or smooth cuticle, stomata located above the other epidermis cells, and characteristic long, twisted non-glandular trichomes. The densely growing non-glandular trichomes may have a relaxing function, preventing clumping of mixed raw materials in herbal mixtures. The groups of bioactive compounds, polyphenols, terpenes, lipids, proteins, and carbohydrates, were identified in the *R. idaeus* glandular trichomes using histochemical assays. Given the bioactivity as well as antimicrobial and antioxidant activity of the compounds, the leaves of this species can be a source of bioactive compounds. The *R. idaeus* leaves are classified as hypostomatic and bifacial. At the ultrastructure level of the leaf cells of the analysed cultivars, the highest concentrations were detected in the case of Na, Ca, Se, and Mo. While K, Ca, Mg, P, and Fe were the dominant elements in the leaf dry mass of the analysed cultivars. Infusions from raspberry leaves are safe for health in terms of the Cd and Pb concentrations, well below the permissible level. Raspberry leaves can be a valuable raw material for preparation of infusions due to the high content of nutrient elements.

Further investigations should be continued to search for bioactive compounds with antiviral, antibacterial, antifungal, antineoplastic, and anti-inflammatory activities in the leaves of the new varieties of *Rubus idaeus*, which adapt well to specific areas of cultivation. The challenge is to apply environmental modifications as well as agrotechnical and genetic engineering methods for modification of the composition of glandular secretions and thus improve the quality of the raw material.

### Sources of funding

The research was supported by the Ministry of Science and Higher Education of Poland in part of the statutory activities of University of Life Sciences in Lublin.

### Contributions by the authors

M.Ch. is responsible for the conceptualization, microscopic observations, writing the original draft, and revision and editing of the manuscript. M.Ch. and M.K. are responsible for the data curation, formal analysis, investigation, and methodology.
